# Social Groups Prioritize Selective Attention to Faces: How Social Identity Shapes Distractor Interference

**DOI:** 10.1371/journal.pone.0161426

**Published:** 2016-08-24

**Authors:** Gewnhi Park, Jay J. van Bavel, LaBarron K. Hill, DeWayne P. Williams, Julian F. Thayer

**Affiliations:** 1 Department of Psychology, Azusa Pacific University, Azusa, California, United States of America; 2 Department of Psychology, New York University, New York, New York, United States of America; 3 Department of Psychiatry and Behavioral Sciences, Duke University Medical Center, Durham, North Carolina, United State of America; 4 Department of Psychology, the Ohio State University, Columbus, Ohio, United States of America; University of Bologna, ITALY

## Abstract

Human faces automatically attract visual attention and this process appears to be guided by social group memberships. In two experiments, we examined how social groups guide selective attention toward in-group and out-group faces. Black and White participants detected a target letter among letter strings superimposed on faces (Experiment 1). White participants were less accurate on trials with racial out-group (Black) compared to in-group (White) distractor faces. Likewise, Black participants were less accurate on trials with racial out-group (White) compared to in-group (Black) distractor faces. However, this pattern of out-group bias was only evident under high perceptual load—when the task was visually difficult. To examine the malleability of this pattern of racial bias, a separate sample of participants were assigned to mixed-race minimal groups (Experiment 2). Participants assigned to groups were less accurate on trials with their minimal in-group members compared to minimal out-group distractor faces, regardless of race. Again, this pattern of out-group bias was only evident under high perceptual load. Taken together, these results suggest that social identity guides selective attention toward motivationally relevant social groups—shifting from out-group bias in the domain of race to in-group bias in the domain of minimal groups—when perceptual resources are scarce.

## Introduction

The human brain is “truly social,” which is to say specialized for group living [1, 2]. Living in groups confers numerous benefits, including the fulfillment of many basic psychological needs [3–6]. The value humans place on group membership is illustrated by the fact that people form groups in every culture on earth [7] and readily form groups under the most trivial of circumstances [8]. People develop an inherent understanding that they belong to a certain group, including the value or significance of this group and the relationship and associations that they share with the group and its members—known as social identification [9, 10]. In this research, we examined when and how social identity impacts one’s capacity of controlling selective attention, critical for goal-directed behavior

### Social identity shapes attention

Social identities can shape a wide range of social and cognitive processes, including the interpretation of social events [11], face memory [12], and visual attention [13]. For example, extensive research has found that racial out-group members often capture one’s attention [14–16]. One recent study examined the effects of racial out-group on *inattentional blindness—*the inability to consciously perceive goal-irrelevant stimuli while attending to goal-relevant stimuli [17]. In a classic demonstration of inattentional blindness, participants were presented with a video clip in which two teams were tossing balls and instructed to count how many times the ball was passed among one of the teams. Fewer than half of the participants reported seeing a person wearing a gorilla suit walk through a scene [18]. Evidence suggests that certain social groups can disrupt inattentional blindness. For instance, White participants were more likely to notice a Black compared to a White interloper, thereby attenuating inattentional blindness [19]. In other words, racial out-group members attracted the attention of White perceivers.

Although racial out-group faces often attract attention and activate stereotypes and evaluations, these biases can be regulated among participants who have both the motivation and cognitive resources (for review see [20]; see also [21]). For example, when White participants have a motivation to pay attention to racial in-group members, such as finding a romantic partner or a friend, they are more likely to notice a White interloper [19]. As such, the motivation and interpersonal goals can override the effects of race, allowing people to employ more egalitarian visual attention. However, controlling the impact of racial out-group biases often comes with high cognitive cost [22]. For instance, when White participants who were asked to perform the Stroop color-naming task following an interracial interaction, they showed impaired task performance after an interracial interaction compared to a same-race interaction [23]. According to the authors, the interracial interaction required the active inhibition of stereotypes, which diminished cognitive resources on the subsequent Stroop task [23]. Similarly, successful goal-directed behavior in most selective attention tasks requires the inhibition of task-irrelevant distractors [24, 25]. As such, controlling or regulating attention and behavior around racial out-group members may require cognitive or perceptual resources.

An alternative strategy for regulating attention is through activating different social identities. Previous research has indicated that social identification is dynamic and varies according to the social context [26, 27]. For instance, merely assigning people to a mixed-race group can elicit a preference for and greater attention to in-group members, regardless of their race [28, 29]. Thus, while prejudice and stereotypes often contribute to intergroup bias in the race domain, identifying with a group—however minimal—can override the effects of race on cognition and behavior. Moreover, participants assigned to these minimal groups often show in-group favoritism, rather than out-group derogation (see [30]). In minimal group contexts, the out-group is often treated as irrelevant or ignored [31]. In-group bias, then, can direct attentional resources toward identity relevant stimuli. Research has yet to examine how these social identities can influence selective attention—that is, how social identity guides attention when perceptual resources are scarce.

### Selective Attention and Cognitive Control

Selective attention refers to one’s ability to focus on motivationally-relevant stimuli while ignoring distractors [32, 33]. According to the Load Theory of Selective Attention and Cognitive Control (henceforth referred to as *Load Theory*), perceptual load determines when selection takes place (see [34]). According to Load Theory, task-relevant stimuli and task-irrelevant distractors compete for limited processing resources [34]. Under low load, when processing task-relevant information is less demanding, spare processing capacity is available to process distractors irrelevant to a goal-directed task. However, under high load, when the amount of task-relevant information is high or the goal-directed task is demanding, limited processing capacity is exhausted, leaving fewer processing resources to process irrelevant distractors [35]. Thus, perceptual load plays an important role in selective attention.

Successful goal-directed behavior in most selective attention tasks requires the inhibition of task-irrelevant distractors [24, 25]. Thus, to effectively perform a task, top-down attentional control must be exerted to facilitate the perceptual processing of task-relevant stimuli and to inhibit the processing of task-irrelevant distractors [24, 36]. However, due to the prioritization of racial out-group faces, even greater attentional control must be exerted to inhibit the processing of task-irrelevant racial out-group face distractors and focus on task-relevant stimuli. Thus, the availability of perceptual resources plays an important role in determining the extent to which top-down attentional control is exerted to inhibit racial out-group face distractors. As such, it may be easier to focus on task-relevant stimuli and inhibit attention to irrelevant racial out-group face distractors under low load when there are ample resources available to permit top-down attentional control. In contrast, it may more difficult to focus on task-relevant stimuli and inhibit attention to irrelevant racial out-group face distractors under high load when there are limited resources available to permit top-down attentional control. As a result, people may show impaired task performance with racial out-group face distractors under perceptual load.

Naturally, the impact of social identity on selective attention should hinge on the particular social identity in question as well as the broader social context. For instance, when people are motivated to engage with in-group members [10, 12], in-group face distractors may produce greater interference effects under high load. Previous research suggests that assigning people to minimal groups imbues in-group members with affective value (see [37]), which then leads to stronger preferences for processing in-group relative to out-group faces [38]. For example, people showed superior recognition [12] and greater activity of the fusiform face area (FFA)—a brain region involved in face perception and recognition—in response to minimal in-group relative to out-group faces (see [31]). Thus, in-group face distractors may be prioritized for selective attention in some contexts.

### Overview

The current research was designed to clarify whether and how social identities shape selective attention. In Experiment 1, White and Black participants were asked to perform a letter detection task in which target letters were superimposed on either Black versus White distractor faces. Under low perceptual load, ample resources were available to exert top-down attentional control to effectively inhibit identity-driven attentional biases and complete the task. Therefore, the Black versus White distractor faces should result in no differential distractor interference under low perceptual load. In contrast, under high perceptual load, fewer perceptual resources were available to exert top-down attention control to inhibit or override out-group bias. We therefore predicted that White participants would show impaired task performance in trials with Black compared to White distractors, and Black participants would show impaired task performance in trials with White compared to Black distractor faces under high load. This experiment would provide evidence that racial out-group distractor faces interfere with selective attention when perceptual resources are scarce.

In Experiment 2, we examined whether assignment to an arbitrary group would produce greater interference effects of in-group face distractors under high perceptual load. We randomly assigned photos of mixed-race faces to a pre-existing in-group or out-group during a learning phase, and then examined the interference effect of these faces under high and low perceptual load. We predicted that participants would show impaired task performance in trials with in-group compared to out-group distractors.

## Experiment 1: Racial Groups and Selective Attention

In Experiment 1, we examined the effects of Black and White male distractor faces on a target detection task under high and low perceptual load. Black and White participants were asked to detect a target from letter strings superimposed on either White or Black face distractors. We predicted that the race of participants and distractor faces would moderate the load effect in a target detection task. Specifically, we hypothesized that participants would be able to inhibit attention toward racial out-group distractors under low perceptual load when greater perceptual resources are available to recruit top-down attentional control which is critical for overriding racial bias (see also [39]). However, racial out-group face distractors may capture one’s attention and lead to greater distractor interference when perceptual resources are unavailable. That is, White participants will be more distracted by Black than White male face distractors and Black participants by White than Black male face distractors under high perceptual load.

### Method

#### Participants

Fifty-four undergraduate students completed the study for partial course credit. There were 31 White participants, but the behavioral data from three White participants were lost due to a computer error, which yielded 28 White participants (20 female; mean age = 19). There were 23 Black participants (18 female; mean age = 21).

#### Ethics statement

Participants received a written informed consent form prior to participating in the study, and all the experiments were reviewed and approved by the Ohio University Institutional Review Board (IRB): Approval number: 2008B0053.

#### Procedure

**Fig 1:**
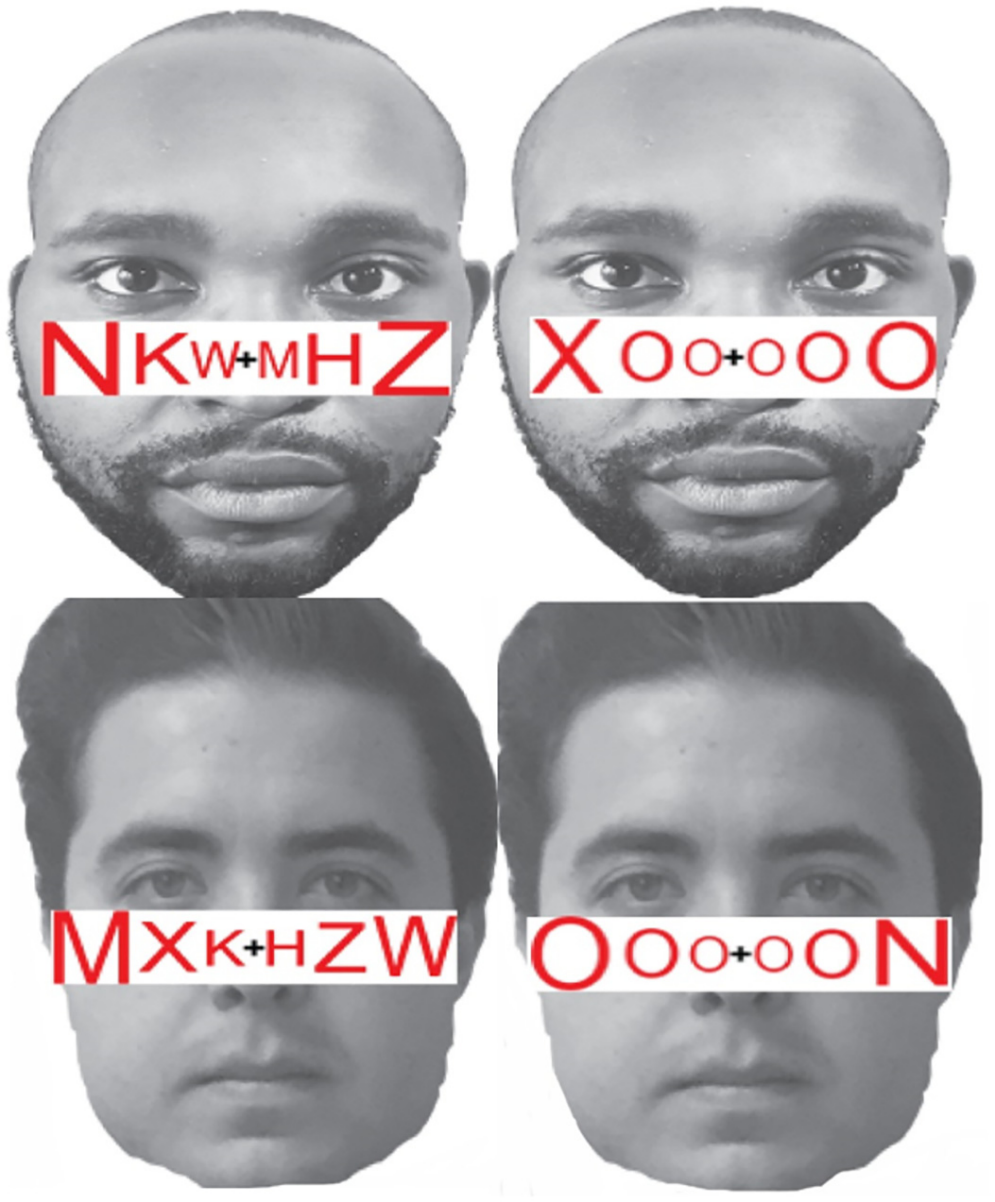
Example stimuli. A string of six letters was superimposed on Black and White male faces. Under high perceptual load, letter strings consisted of one target letter and five non-target letters arranged in random order. Under low perceptual load, letter strings consisted of five O’s and one target letter (X or N). Blackfaces under high perceptual load (top left). Black faces under low perceptual load (top right). White faces under high perceptual load (bottom left). White faces under low perceptual load (bottom right).

Participants completed a letter detection task in which they saw a series of letter strings and were instructed to identify whether each letter string contained an “X” or an “N” by pressing the corresponding keys on the keyboard as quickly and accurately as possible. Participants completed this task under high or low perceptual load, with Black or White distractor faces in the background. During each trial, participants saw a face with the middle of the nose at fixation and a string of six letters, written in red, superimposed across this middle point (see Fig 1).

**Fig 2:**
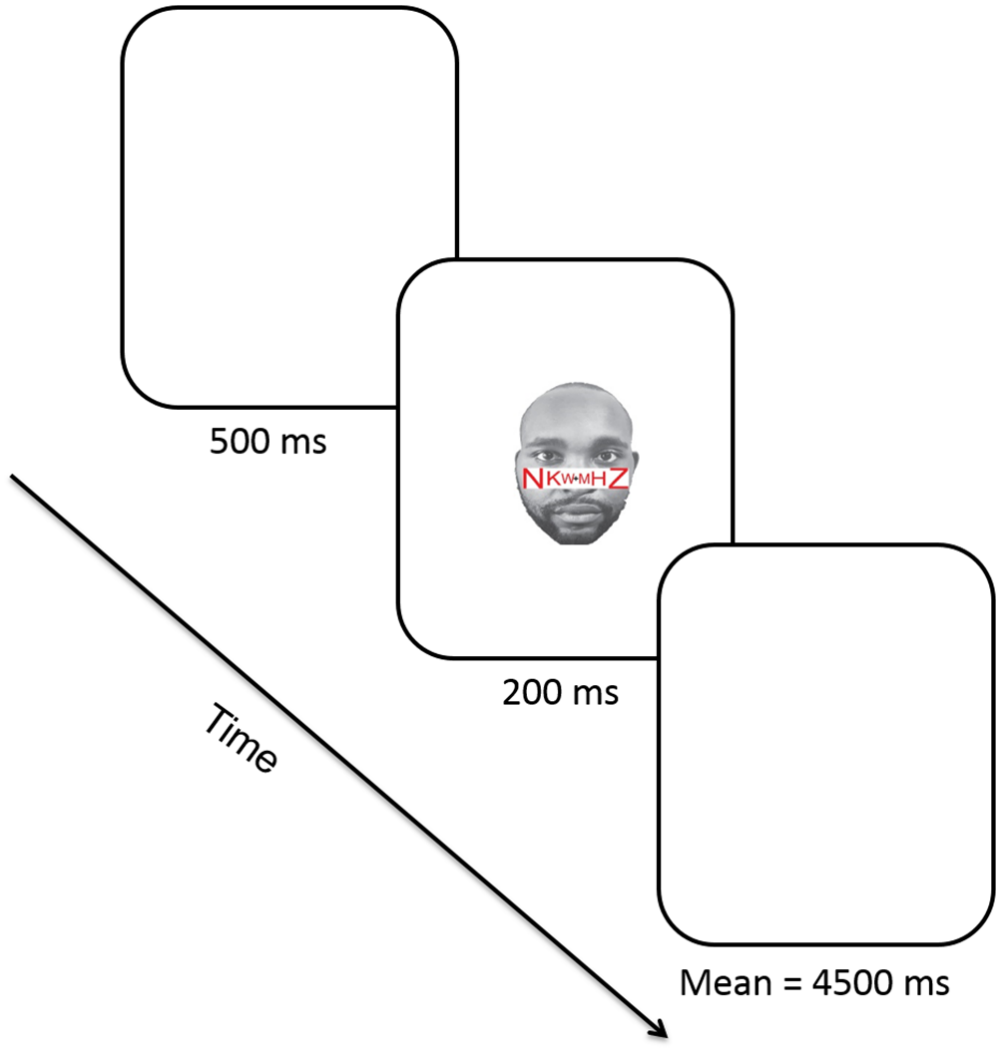
Example of experimental sequence. The fixation cross was presented for 500 ms and followed by the display with a string of six letters superimposed on a face for 200 ms. The interstimulus interval was randomly generated with a mean of 4500 ms. Stimuli are not drawn to scale.

There were alternating blocks of the high- or low-load letter task (starting condition counterbalanced). Each block started with 12 practice trials with just the letter strings presented, followed by 48 experimental trials in random order (96 trials in total). Each experimental trial began with a fixation cross for 500 ms, followed by the display of the string of six letters superimposed on a face for 200 ms (see Fig 2). The interstimulus intervals were randomly generated with a mean of 4500 ms. Participants were also told to ignore the faces throughout the task. When participants failed to respond, they received feedback indicating that they had not responded in time. Response accuracy and RT were recorded on each trial. All participants were tested individually.

#### Materials

Each display consisted of a face with the nose centered at fixation and six letters superimposed (see Fig 1; [40, 41]). We employed the letter strings used by Lim and colleagues (2008), in which letter size was adjusted to compensate for the advantage of foveal presentation and the disadvantage of peripheral presentation. The letter string was written in red and enclosed in a White frame. In the *high* perceptual load condition, the letter string comprised one target letter (X or N) and five non-target letters (H, K, M, W, or Z) arranged in random order [40, 41]. In the *low* perceptual load condition, the letter string comprised one target letter (X or N) and five O’s [41] in random order. The combination of target letter identity and position was counterbalanced across four different conditions.

The face stimuli consisted of pictures of six White males and six Black males (adapted from [37]). Faces were converted to grayscale (256 gray levels) and edited to remove background information. The contrast and brightness of each face was adjusted to maintain constancy across faces. Each face measured 6° horizontally and 7.5° vertically against a White background. All materials are available online at: https://osf.io/a9vkn/.

#### Analyses

Accuracy data were organized on a trial-by-trial basis, such that accuracy of each trial was represented as a binary outcome (e.g. 1 = accurate and 0 = error). Reaction times (RT) of less than 150 ms or more than 1200 ms were considered outliers and were excluded (2% of trials; Ratcliff, 1993). All analyses on RT excluded outliers *and* incorrect trials (Ratcliff, 1993). We conducted the generalized linear mixed models using SPSS 22.0.1 (SPSS Inc, Chicago, Illinois) on dichotomous accuracy data. Previous literature that studied the topic (i.e., attentional load) have used ANOVA on accuracy [24, 34, 37, 40, 41]). However, according to Jaeger [42], categorical data such as accuracy data violated, by definition, ANOVA assumptions and could lead to spurious results. Thus, the generalized may serve as a better alternative to analyze accuracy data to avoid spurious results and to increase more power [42]. As fixed variables, we entered three categorical predictors (load, race of participants, and race of distractor faces) along with their interactions, including the three-way interaction. We added by-subject random intercept and slope for all main effects and interactions of three variables (load, race of participants, and race of distractor faces). We selected the binary logistic regression and first-order autoregressive (AR1) for the covariance structure. Separate effect-coded variables were created for race of participants (Black = 1, White = 2), race of distractor face (Black = 1, White = 2), and perceptual load (low = 1, high = 2).

#### Results and Discussion

We first assessed the success of the load manipulation. As predicted, there was a significant load effect, such that participants were less accurate to detect targets during the high load trials (*M* = 77.8%, *SD* = 8.7%) than during the low load trials (*M* = 93.7%, *SD* = 14.3%), providing evidence that we successfully manipulated perceptual load, *F*(1, 4.89) = 272.84, *p* = .001 (see Tables 1 and 2). The trimmed RT were analyzed using linear mixed models (see Table 1 for mean RTs). As fixed variables, we entered three categorical predictors (load, race of participants, and race of distractor faces) along with their interactions, including the three-way interaction. As fixed variables, we entered three categorical predictors (load, race of participants, and race of distractor faces) along with their interactions, including the three-way interaction. We added by-subject random intercept and slope for all main effects and interactions of three variables (load, race of participants, and race of distractor faces). We selected unstructured for the covariance structure and restricted maximum likelihood estimation. This pattern was similar for RTs, such that participants were slower under high (*M* = 876 ms, *SD* = 110) in comparison to low (*M* = 602 ms, *SD* = 87) perceptual load, β = 274.96, *SE =* 22.83, *p* < .01 (95% CI 175.10, 374.82). However, there was no interaction (*ps* > .44). This suggests that there is no speed-accuracy trade off. Consistent with previous research, this result provided evidence that perceptual load impaired target detection (see [24, 25, 35]). The data are available online at: https://osf.io/bhukm/.

**Table 1:** Mean Response Accuracy and Reaction Times (in milliseconds), as a Function of Task Load, Race of Participants, and Race of Distractor Face in Experiment 1. Standard Deviations in Parentheses.

Race of participants	Black				White			
Load	High load		Low load		High load		Low load	
Race of distractor faces	Black	White	Black	White	Black	White	Black	White
Response accuracy	77.4 (9.8)	73.4 (10.3)	94.0 (7.2)	95.6 (7.3)	76.6 (9.7)	82.1 (8.5)	92.5 (18.3)	92.5 (18.9)
Trimmed RTs	852.5 (92.7)	836.8 (106.2)	586.1 (89.6)	579.8 (85.2)	905.8 (126.3)	909.9 (126.3)	623.5 (88.1)	620.5 (89.2)

**Table 2:** Results of linear mixed model on Mean Response Accuracy as a Function of Task Load, Race of Participants, and Race of Distractor Face in Experiment 1.

Variable	Beta	SE	t	Significance	95% Confidence Interval	
					Lower	Upper
Intercept	-1.58	0.13	-11.90	< .001*	-1.84	-1.32
Load	-1.65	0.22	-7.39	< .001*	-2.08	-1.21
Race of distractor face	0.39	0.14	2.82	< .01*	0.12	0.66
Race of participants	0.52	0.19	2.74	< .01*	0.15	0.89
Load × Race of participants	-0.42	0.32	-1.32	0.19	-1.05	0.20
Load × Race of distractor face	-0.35	0.31	-1.14	0.25	-0.96	0.25
Race of participant × Race of distractor face	-0.61	0.20	-3.10	< .10*	-1.01	-0.22
Load × Race of distractor face × Race of participants	0.87	0.44	2.00	< .05*	0.01	1.73

Note:

* indicates statistical significance.

**Fig 3:**
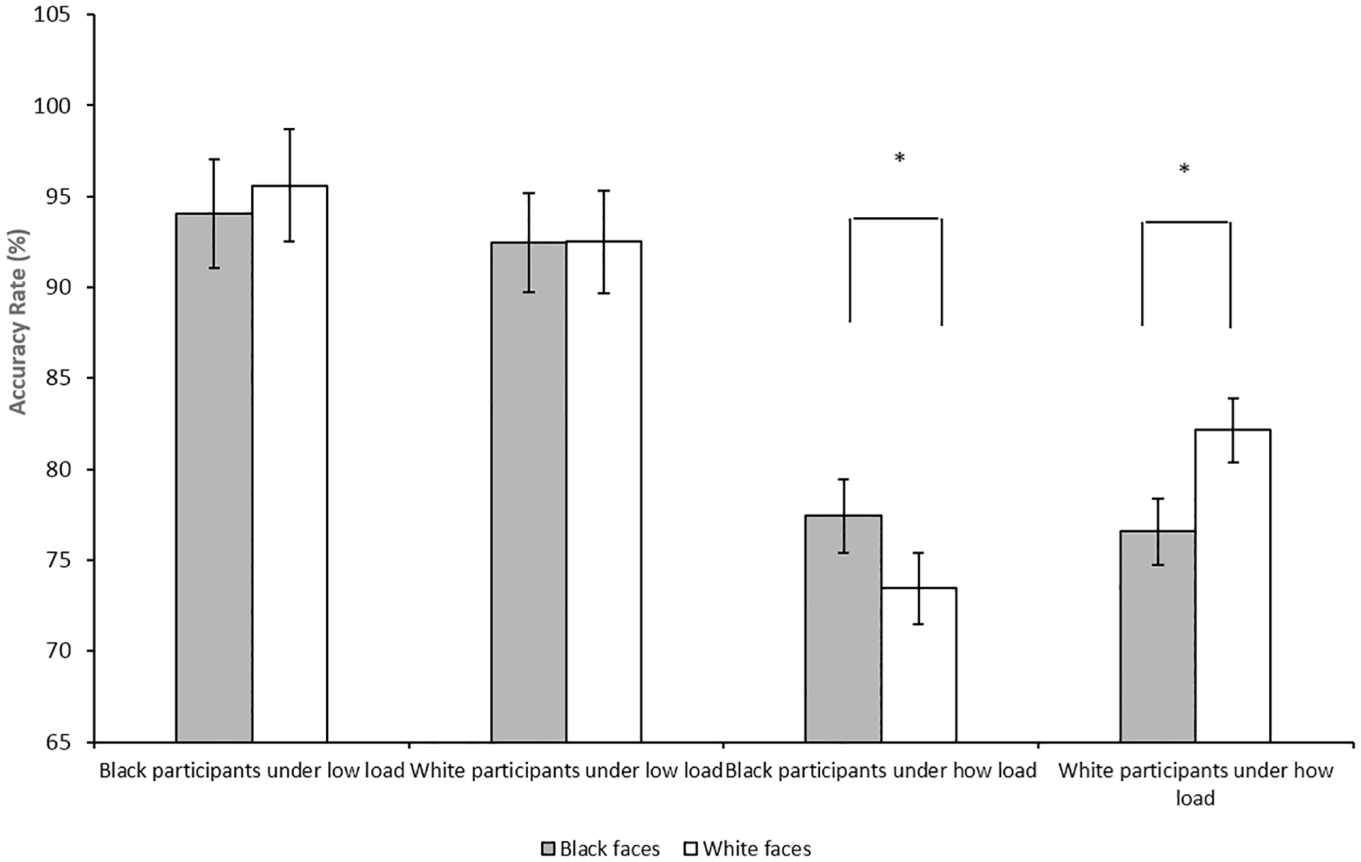
Accuracy rates and standard errors as a function of perceptual load and race of participants and distractor faces in Experiment 1. Black participants were less accurate on trials with other-race (White) compared to own-race (Black) face distractors under high perceptual load; however, there was no difference in accuracy between other-race (White) and own-race (Black) face distractors under low perceptual load. Likewise, White participants were less accurate on trials with other-race (Black) compared to own-race (White) face distractors under high perceptual load; however, there was no difference in accuracy between other-race (Black) and own-race (White) face distractors under low perceptual load. It should be noted that the analysis was presented in Fig 3 was based on the raw means, not parameters from the multi-level models. Error bars = standard errors. Note: * *p* ≤ .05.

We next sought to investigate whether the interaction between the race of participants and distractor faces on accuracy depends on perceptual load. If White participants show reduced performance to Black distractor faces and Black participants to White distractor faces under high perceptual load, it would provide evidence that distractor interference depends not only on the cognitive processes associated with social identity, but also on perceptual load. Consistent with the hypothesis, we found a significant 3-way interaction between load, race of participants, and race of distractor faces on accuracy, *F*(1, 4.89) = 272.84, *p* = .001 (see Fig 3).

To directly test the hypotheses, we examined the main effects and interaction between race of participants and race of distractor faces for high and low load, separately. Consistent with the hypothesis, there was a significant interaction between race of participants and race of distractor face on accuracy under high perceptual load, β =—0.61, *SE =* 0.20, *p* < .01 (95% CI—.99,—.22). Black participants were less accurate in trials with White (*M* = 73.4%, *SD* = 10.3%) than Black distractor faces (*M* = 77.4%, *SD* = 9.8%) under high perceptual load, *t*(22) = 2.68, *p* < .02, *d* = .40. In contrast, white participants were less accurate to Black (*M* = 76.6%, *SD* = 9.7%) than White distractor faces (*M* = 82.1%, *SD* = 8.5%) under high perceptual load, *t*(27) = -3.49, *p* < .01, *d* = 0.61. However, there was no evidence of differential distraction to Black or White distractor faces under low load (*p* > .61). Thus, consistent with our hypothesis, participants were able to inhibit the processing of racial out-group face distractors under low perceptual load when greater perceptual resources are available to override racial out-group bias (see also [39]. However, racial out-group face distractors may capture one’s attention and lead to greater distractor interference under high load when perceptual resources are limited.

### Conclusions

These results suggest that racial out-group faces may have produced greater interference effects under high load when cognitive sources were scarce to inhibit attention to racial out-group distractor faces. In contrast, there was no evidence that participants were more distracted by racial out-group compared to in-group distractor faces under low perceptual load, when participants had more perceptual resources available to control their attention. Therefore, our study suggests that effects of race on selective attention under high perceptual load hinge upon social identify.

## Experiment 2: Minimal Groups and Selective Attention

To examine the malleability of this pattern of racial bias in selective attention, we manipulated group membership in Experiment 2. Specifically, we assigned participants to mixed-race minimal groups to see if this seemingly trivial group membership would replace the interference effects of racial out-group on task performance under load. Although many scholars have described racial out-group bias as automatic and inevitable [43, 44], there is good reason to believe that making alternative group identities salient can override racial bias (see [10] for a discussion). Thus, although the first experiment suggested that out-group faces might be most distracting under high perceptual load, the minimal group assignment would lead to preferential processing for in-group faces, thereby resulting in greater distractor interference of in-group face distractors under high perceptual load [10, 31, 37]. To investigate this issue, we randomly assigned people to a minimal in-group or out-group (group membership was indicated by the background color of each photo) during a learning phase, and then used these faces as distractors in a target detection task to measure distractor interference. It was expected that the pattern of racial bias observed in Experiments 1 would be eliminated, and in-group faces would produce greater distractor interference effects under high perceptual load.

### Method

#### Participants

Forty-five undergraduate students completed the study for partial course credit (24 female; mean age = 20). Of the participants, one was American Indian, 20 were White, 15 were Asian, two were Hispanic, two were African-American, and five did not provide a specific race. It should be noted that the results were virtually identical when we excluded the two Black participants.

#### Procedure

**Group Assignment:** Participants were instructed that they were in a study learning about groups and that they would be randomly assigned to the blue or green team (adapted from [31, 37]). They were instructed to remember the members of their own team and the other team before moving to the next phase of the study. Then, participants were presented with 12 faces (six in-group and six out-group faces) for 2000 ms each on either a blue or green background. The 12 faces used in the letter detection task in Experiment 1 were used in the group assignment task, and faces were randomly assigned to the teams. Six in-group and six out-group faces consisted of three Black and three White faces each. This design ensured that participants would be equally likely to see each face as an in-group or out-group color denoted group membership (in-group/out-group) and was counterbalanced across participants. After the completion of the group assignment task, participants were asked to perform the letter detection task identical to Experiment 1.

**Letter Detection:** The stimuli, procedure and analyses were identical to Experiments 1. Like Experiment 1, we conducted the generalized linear mixed models using SPSS 22.0.1 (SPSS Inc, Chicago, Illinois) on accuracy data. As fixed variables, we entered three categorical predictors (load, group membership, and race of distractor faces) along with their interactions, including the three-way interaction. We added by-subject random intercept and slope for all main effects and interactions of all three variables (load, group membership, and race of distractor faces). We selected the binary logistic regression and first-order autoregressive (AR1) for the covariance structure. Separate effect-coded variables were created for group membership (in-group = 1, out-group = 2), race of distractor face (Black = 1, White = 2), and perceptual load (low = 1, high = 2).

**Collective Identification:** Participants answered six questions assessing their collective identification with being in the in-group or the out-group using a 6-point Likert scale [10]: “I value being a member of BLUE (GREEN) team”, “I am proud to be a member of the BLUE (GREEN) team”, “Belonging to the BLUE (GREEN) team is an important part of my identity”. Ratings and reaction time were recorded. We computed an index of collective identification *strength* by separately adding the ratings for the three in-group items and subtracting the three out-group items, and an index of *accessibility* by computing the difference of the log-transformed RTs for in-group and out-group ratings [13]. Positive collective identification accessibility scores indicate higher relative accessibility of in-group identification [13, 45]. These items were embedded within a series of other unrelated questionnaires (e.g., measures of the Big 5 personality traits and self-esteem) to help disguise the intent of our experiment. These questionnaires were not analyzed.

### Results

#### Collective identification

Participants reported higher collective identification strength for the in-group (*M* = 9.96, *SD* = 4.42) than the out-group (*M* = 5.58, *SD* = 3.32), identifying more with their in-group than the out-group, *t*(44) = 5.97, *p* < .97, *d* = 1.12. This confirmed that the minimal group manipulation was successful.

#### Letter Detection Task

As in Experiments 1, participants were less accurate to detect targets during the high load trials (*M* = 73.7%, *SD* = 11.4%) than during the low load trials (*M* = 93.8%, *SD* = 9.5%), providing evidence that we successfully manipulated perceptual load, *F*(1, 8.63) = 318.64, *p* < .01 (see Table 3). This pattern was similar for RTs, such that participants were slower under high (*M* = 800 ms, *SD* = 145.9) than low perceptual load (*M* = 537 ms, *SD* = 117.9), β = 262.68, *SE =* 20.95, *p* < .01 (95% CI 220.47, 304.90). However, there was no interaction (*ps* > .44). However, there was no main effect or interaction (*ps* > .14). This suggests that there is no speed-accuracy trade off. The data are available online at: https://osf.io/bhukm/.

**Table 3:** Mean Response Accuracy and Reaction Times (in milliseconds), as a Function of Group Assignment and Task Load in Experiment 2. Standard Deviations in Parentheses.

	High load		Low load	
	Out-group distractor faces	In-group distractor faces	Out-group distractor faces	In-group distractor faces
Response Accuracy	75.5 (11.5)	71.9 (12.6)	93.5 (9.1)	94.0 (10.3)
Trimmed RTs	802.1 (156.4)	798.9 (142.6)	540.5 (119.5)	535.2 (117.0)

Based on previous research [10, 29], we hypothesized that assignment to a mixed-race group would eliminate the interference effects of race on task performance under high perceptual load. Indeed, there was no main effect of race of distractor face on accuracy, *F*(1, 8.63) = 0.07, *p* = .80, nor the interaction between load and race of distractor, *F*(1, 8.63) = 0.06, *p* = .80, suggesting that a mixed-race minimal group manipulation overrode the interference effects of race under high perceptual load (see Table 4). In contrast to Experiment 1, there was no significant interaction between load and race on accuracy, when we analyzed White participants only, β =—0.60, *SE =* 0.34, *p* = .08 (95% CI—1.27, .06).

**Table 4:** Results of linear mixed model on Mean Response Accuracy as a Function of Task Load, Race of Participants, and Race of Distractor Face in Experiment 1.

Variable	Beta	SE	t	Significance	95% Confidence Interval	
					Lower	Upper
Intercept	-1.23	0.14	-9.14	< .0001*	-1.49	-0.97
Group membership	0.26	0.14	1.82	0.07	-0.02	0.54
Load	-1.61	0.18	-9.12	< .0001*	-1.96	-1.26
Race of distractor face	0.07	0.14	0.54	0.59	-0.19	0.34
Load × Group membership	-0.56	0.24	-2.35	< .2*	-1.03	-0.09
Load × Race of distractor face	-0.17	0.23	-0.73	0.47	-0.62	0.29
Race of distractor face × Group membership	-0.14	0.17	-0.82	0.42	-0.47	0.19
Load × Race of distractor face × Group membership	0.42	0.32	1.33	0.18	-0.20	1.04

Note:

* indicates statistical significance.

**Fig 4:**
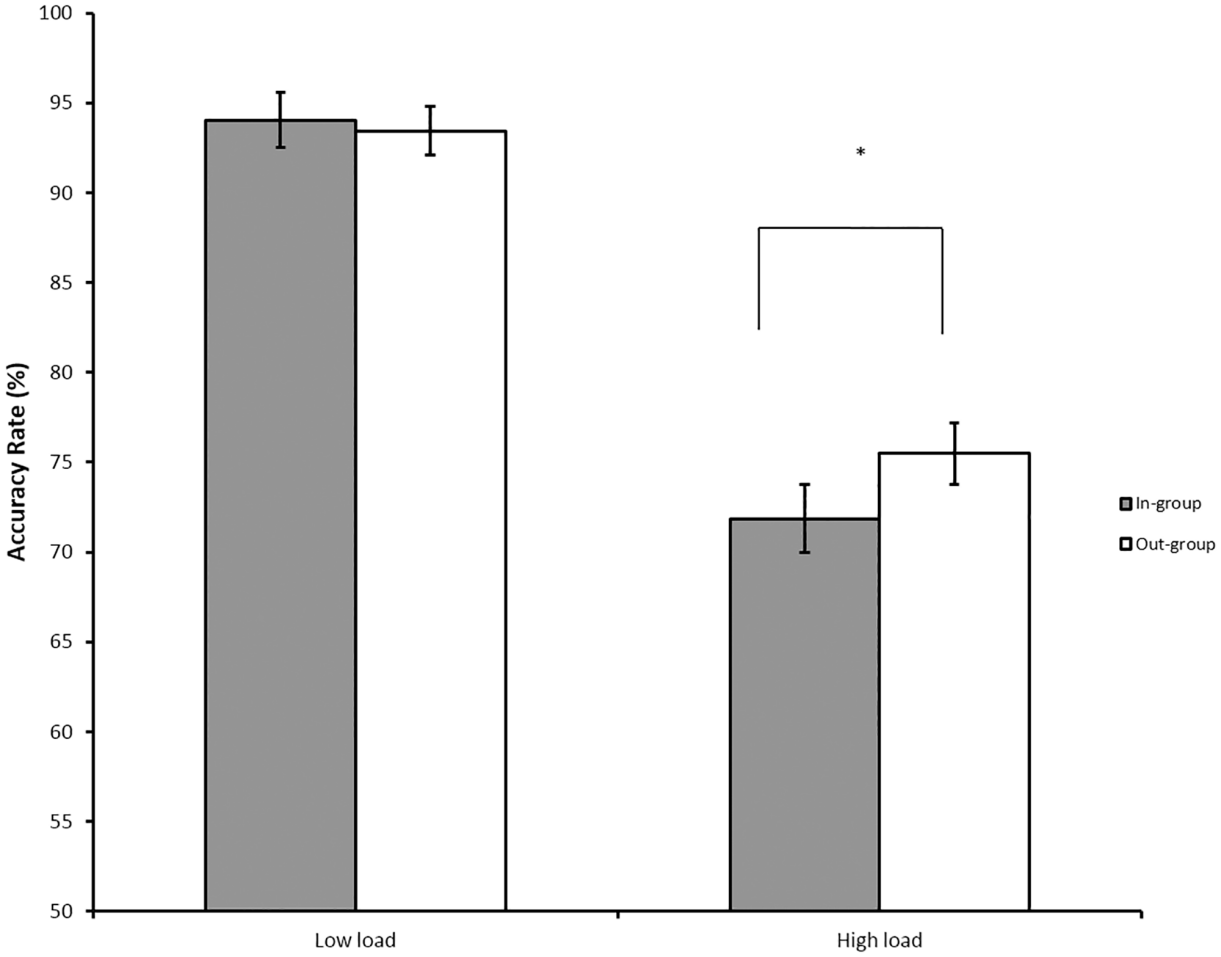
Accuracy rates and standard errors as a function of perceptual load and social identity of distractor faces in Experiment 2. Participants were less accurate on trials with in-group compared to out-group face distractors under high perceptual load; however, they showed no difference under low perceptual load. It should be noted that the analysis was presented in Fig 4 was based on the raw means, not parameters from the multi-level models. Error bars = standard errors. Note: * *p* ≤ .05.

We hypothesized that minimal group assignment would facilitate the processing of in-group faces and become more distracting under high perceptual load. Indeed, there was the significant interaction between group assignment and load, *F*(1, 8.63) = 3.91, *p* < .05, such that participants were less accurate on trials with in-group distractor faces (*M* = 71.9%, *SD* = 12.6%) than with out-group distractor faces (*M* = 75.5%, *SD* = 11.5%) under high perceptual load, *t*(44) = 3.04, *p* < .01, *d* = .30 (see Fig 4). There was no difference under low perceptual load (*p* > .42) and no other interaction proved significant (*ps* > .18). Therefore, random assignment to a mixed-race minimal group not only eliminated any evidence of racial bias, but it also reversed the interference effect of racial out-group faces, such that greater interference effects were observed in response to in-group compared to out-group face distractors under high perceptual load.

#### Correlation between collective identification and distractor interference under high load

**Fig 5:**
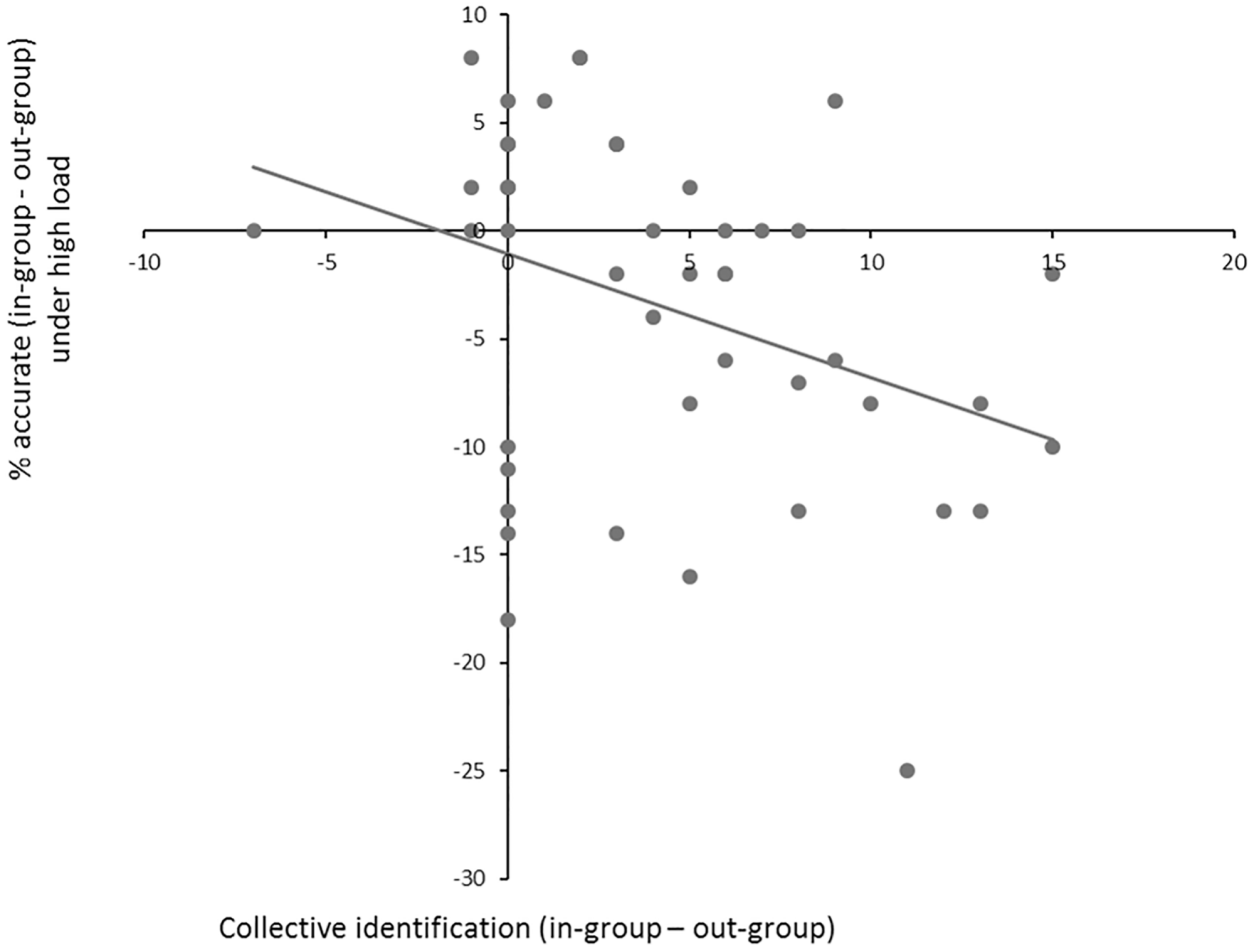
Correlation between collective identification scores (x-axis) and distractor interference towards in-group faces (y-axis) under high perceptual load. Participants with higher relative in-group (versus out-group) identification showed increased distractor interference toward in-group (versus out-group) faces (i.e., decreased accuracy when in-group faces were used as distractors). *r*(45) = -.36, *p* < .02.

Additionally, we hypothesized that participants with higher relative in-group identification accessibility would show increased distractor interference effects of in-group faces under high perceptual load (see [13]). As predicted, participants with higher relative in-group (versus out-group) accessibility showed decreased accuracy to detect targets in trials with in-group face distractors (see Fig 5): *r*(45) = -.36, *p* < .02. In other words, participants with more accessibility in-group (versus out-group) identities showed increased interference toward in-group (versus out-group) distractor faces.

### Discussion

We predicted that selective attention would be preferentially drawn to in-group members in minimal groups (see [31]). Indeed, the minimal group assignment resulted in greater interference effects of in-group compared to out-group face distractors under high perceptual load. This result may provide evidence that the minimal group assignment leads to the preferential processing (e.g., attentional capture) of in-group face distractors, thereby leading to greater distractor interference under high perceptual load. It is difficult to attribute the results to facial features of stimuli because this design ensured that participants would be equally likely to see each face as an in-group or out-group color denoted group membership (in-group/out-group) and was counterbalanced across participants. Furthermore, the magnitude of in-group bias in distractor interference was correlated with the accessibility of in-group identification—directly linking identification to attention.

## General Discussion

We explored whether social identities shape selective attention to perform effective goal-directed behavior. Furthermore, we examined how the influence of social identities on selective attention may depend on whether perceptual resources are adequate or scarce, which can be manipulated by perceptual load (see [34]). As expected, participants exhibited greater interference effects of racial out-group relative to racial in-group face distractors under high perceptual load, when fewer processing resources were available (Experiment 1). However, the race of distractor faces did not interfere with task performance under low perceptual load. In contrast, participants assigned to minimal groups revealed greater interference effects of in-group relative to out-group distractor faces under high perceptual load, which provides evidence of the malleability of racial bias in selective attention (Experiment 2).

The current research provides strong evidence that social identifies shape selective attention to perform effective goal-directed behavior. Moreover, the effects of identity on selective attention appear to depend on the availability of perceptual resources. Under low perceptual load when processing task-relevant information was relatively easy, spare perceptual resources could be used to process racial out-group distractor faces, yet there were still enough perceptual resources available to exert top-down attentional control to inhibit attentional biases toward racial out-group distractor faces. We speculate, however, that the frontal-parietal networks involved in controlled processing were successfully recruited under low perceptual load to inhibit differential attention to racial out-group faces and focus on the letter detection task (see [46]). Under high perceptual load, the same networks may have been recruited for the task, reducing the perceptual resources available to diminish the differential attention to racial out-group faces [24, 35]. Thus, while racial out-group faces may capture attention in both conditions (relative to racial in-group faces), the present research suggests that participants are capable of recruiting top-down attentional control to inhibit their attentional bias toward racial out-group faces in the low perceptual load condition. However, future research should directly examine the psychological factors that may account for this pattern of results (e.g., [47]) to determine whether inhibitory processes account for the differential response to racial out-group faces in the high and low load conditions. This research extends the previous research on racial out-group faces and attention by showing that attentional bias (e.g., [4, 14–16]) may be inhibited when participants have sufficient perceptual resources (see also [48]).

The current research extends the Load Theory proposed by Lavie and her colleagues [34, 35]. Lavie and her colleagues [34, 35] have conceptualized a load-dependent locus of selective attention: the distractor interference effect on selective attention depends on the availability of processing resources under load [25, 34, 35, 49]. According to the theory, distractors significantly interfere with task performance under low perceptual load whereas their effect is diminished under high load. The results in our experiments suggest that social identity may shape distractor inference. Other studies have identified similar evidence of distractor interference under high perceptual load. For instance, when famous faces are used as distractors, participants’ ability to detect a target is significantly diminished—regardless of the level of perceptual load [50], thereby providing evidence that load does not moderate the distractor interference of socially significant stimuli. Furthermore, a recent study reveals that individual differences play a role in task performance under load [25]: people with good self-regulatory function, indexed by higher resting cardiac vagal tone, were faster in trials with neutral distractors under high perceptual load. Taken together, these studies suggest that load theory needs to be revised to account for the influence of social and motivational concerns on behavior under perceptual load.

Our results may appear to contradict previous research in which participants did not activate categorical knowledge under high load [51]. For example, Gilbert & Hixon [51] presented participants with word fragments, some of which could be resolved by using stereotypic words that describe Asian Americans, such as shy or rice, while being exposed to either a White or Asian female assistant. Participants with *low* working memory load generated greater stereotypic responses in the presence of an Asian assistant compared to a White assistant. In contrast, participants with *high* working memory load were not influenced by the race of the assistant. There are a number of important differences that may account for the apparent discrepancy between these results and present research. First, Gilbert & Hixon [51]) manipulated the amount of *working memory* load whereas we manipulated *perceptual* load in an attentional task. Although greater distractor interference effects are typically observed under high perceptual load, the opposite is true for working memory load [35, 52]. Second, presenting a series of faces in which the only obvious difference was race and in which the faces were equally likely to be Black and White—a notable departure from the base rates in the local population—may have made race salient.

It is possible to interpret the results in terms of arousal induced by varying group distractors. Racial out-group distractors and minimal in-group distractors both could have elicited both arousing emotion, which may lead to a detrimental performance under high load, when fewer perceptual resources are available. In fact, it has been well know that both motivationally and socially relevant stimuli elicit the activation of neural structures that underlie attention [37, 46, 53]. However, their effects on selective attention under load appear to be different. Previous research has indicated that participants were more accurate on trials with fearful compared to neutral faces under high load [24, 25]. Conversely, participants were less accurate on trials with socially relevant stimuli (e.g., racial out-group or minimal in-group faces). Motivationally relevant stimuli (e.g., fearful faces) may signal a sign of threat and alert people to look for its source in the environment, which may have allowed people to identify targets better. Future work should attempt to differentiate the effect of arousal caused by emotionally arousing (e.g., fearful faces) and socially relevant stimuli on selective attention.

There are some limitation of the current research. First of all, the current research used only male faces. Therefore, future work is necessary to see if the effects extend to other stimuli, including female faces. Secondly, Experiment 2 included Asian, Hispanic and potentially unspecific race participants. Although we did not observe the effect of race of distractor faces among White participants in Experiment 2 (see foot note 7), which was contrast to Experiment 1, it is still possible that the mixed race make-up of the participants not the minimal-group assignment may cause the absence of a main effect of race of distractor faces. Therefore, future work should include only Black and White participants to eliminate the potential effect of mixed race make-up of participants.

## Conclusions

We live in a world where we frequently interact and work with members of various groups to achieve our goals. As such, we are constantly exposed to an environment where social information, especially social identity, can easily and often become salient. It is important to know when this information can become a distraction that prevents one from maximizing goal-directed performance. The current research provides evidence that social identity plays an important role in one’s ability for controlling selective attention when perceptual resources are limited. Our research provides evidence that social identity can prioritize selective attention even at the costs of distracting us from other goal-directed behavior.
