# Correction: Social Groups Prioritize Selective Attention to Faces: How Social Identity Shapes Distractor Interference

**DOI:** 10.1371/journal.pone.0164909

**Published:** 2016-10-12

**Authors:** Gewnhi Park, Jay J. Van Bavel, LaBarron K. Hill, DeWayne P. Williams, Julian F. Thayer

The second author’s name is spelled incorrectly. The correct name is: Jay J. Van Bavel. The correct citation is: Park G, Van Bavel JJ, Hill LK, Williams DP, Thayer JF (2016) Social Groups Prioritize Selective Attention to Faces: How Social Identity Shapes Distractor Interference. PLoS ONE 11(8): e0161426. doi:10.1371/journal.pone.0161426

Additionally, there are typos in the x-axis in Fig 3. It should read “high load,” not “how load.” Please view the corrected Fig 3 here.

**Fig 3:**
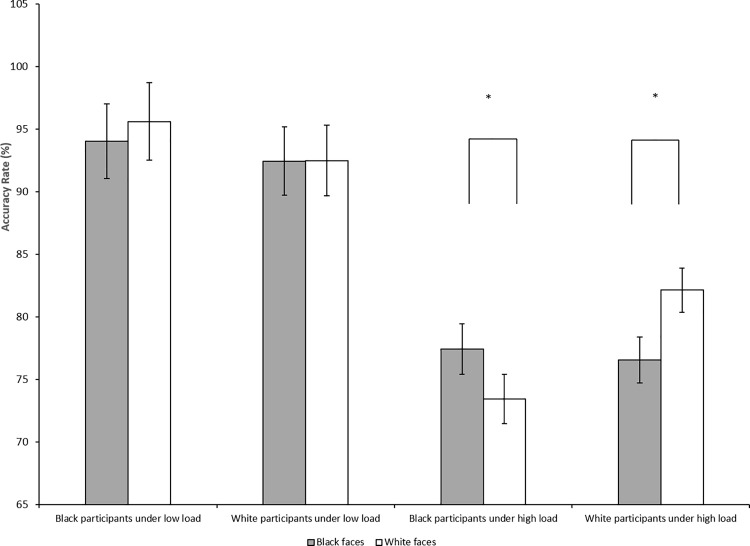
Accuracy rates and standard errors as a function of perceptual load and race of participants and distractor faces in Experiment 1. Black participants were less accurate on trials with other-race (White) compared to own-race (Black) face distractors under high perceptual load; however, there was no difference in accuracy between other-race (White) and own-race (Black) face distractors under low perceptual load. Likewise, White participants were less accurate on trials with other-race (Black) compared to own-race (White) face distractors under high perceptual load; however, there was no difference in accuracy between other-race (Black) and own-race (White) face distractors under low perceptual load. It should be noted that the analysis was presented in Fig 3 was based on the raw means, not parameters from the multi-level models. Error bars = standard errors. Note: * p ≤ .05.
